# Oasis Malaria, Northern Mauritania^1^

**DOI:** 10.3201/eid2502.180732

**Published:** 2019-02

**Authors:** Jemila Deida, Rachida Tahar, Yacoub Ould Khalef, Khadijetou Mint Lekweiry, Abdoullah Hmeyade, Mohamed Lemine Ould Khairy, Frédéric Simard, Hervé Bogreau, Leonardo Basco, Ali Ould Mohamed Salem Boukhary

**Affiliations:** Unité Mixte de Recherche 216, Institut de Recherche pour le Développement, Université Paris 5, Sorbonne Paris Cité, Paris, France (J. Deida, R. Tahar);; Université de Nouakchott Al-Aasriya, Nouakchott, Mauritania (J. Deida, K. Mint Lekweiry, A. Ould Mohamed Salem Boukhary);; Ministry of Health, Nouakchott (Y. Ould Khalef, M.L. Ould Khairy); Ministry of Health, Atar (A. Hmeyade);; Maladies Infectieuses et Vecteurs: Ecologie, Génétique, Evolution et Contrôle, Institut de Recherche pour le Développement–Centre National de Recherche Scientifique– Université Montpellier, Montpellier, France (F. Simard);; Institut de Recherche Biomédicale des Armées, Marseille (H. Bogreau);; Centre National de Référence du Paludisme, Marseille (H. Bogreau);; Aix Marseille Université, Institut de Recherche pour le Développement, Assistance Publique–Hôpitaux de Marseille, Service de Santé des Armées, Vecteurs–Infections Tropicales et Méditerranéennes, Marseille, France (H. Bogreau, L. Basco, A. Ould Mohamed Salem Boukhary)

**Keywords:** malaria, *Plasmodium vivax*, parasites, oasis, Atar, Sahara, Mauritania, vector-borne diseases

## Abstract

A malaria survey was conducted in Atar, the northernmost oasis city in Mauritania, during 2015–2016. All febrile patients in whom malaria was suspected were screened for malaria by using rapid diagnostic testing and microscopic examination of blood smears and later confirmed by PCR. Of 453 suspected malaria cases, 108 (23.8%) were positive by rapid diagnostic testing, 154 (34.0%) by microscopic examination, and 162 (35.7%) by PCR. Malaria cases were observed throughout the year and among all age groups. *Plasmodium vivax* was present in 120/162 (74.1%) cases, *P. falciparum* in 4/162 (2.4%), and mixed *P. falciparum*–*P. vivax* in 38/162 (23.4%). Malaria is endemic in northern Mauritania and could be spreading farther north in the Sahara, possibly because of human-driven environmental changes. Further entomologic and parasitologic studies and monitoring are needed to relate these findings to major *Anopheles* mosquito vectors and to design and implement strategies for malaria prevention and control.

Malaria is one of the major reasons for seeking healthcare in public health facilities in Mauritania. In 2016, among an estimated population of 3,537,368, malaria incidence was 78/1,000 persons at risk (*1*) and malaria-associated mortality was 30 deaths/100,000 persons (*2*). The northern limit where malaria transmission occurs in Mauritania is not well defined. Sporadic suspected cases of malaria were reported in the oasis setting of Atar in the early 2010s, and the first *Plasmodium vivax* cases were confirmed by microscopic examination in 2012, but PCR was not performed for further confirmation (A. Ould Mohamed Salem Boukhary, unpub. data). Therapeutic efficacy of chloroquine to treat *P. vivax* malaria was also evaluated in 2013 in Atar (*3*). In that study, patient screening and recruitment were performed during only 1 month, followed by a 28-day follow-up period. Here, we report results of a follow-up study in which we conducted a longitudinal survey in Atar to establish baseline data on malaria burden in this Saharan zone of Mauritania.

## Materials and Methods

### Study Site

**Figure 1:**
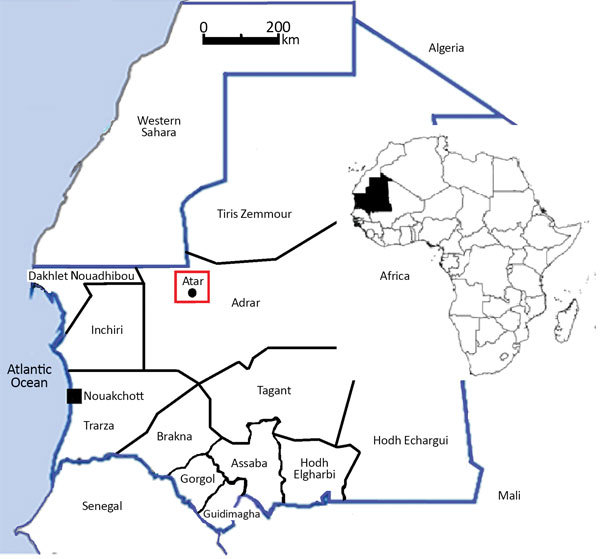
Study site for investigation of malaria in Mauritania (red box). Twelve provinces and Nouakchott (the capital city) are also shown. Inset map shows location of Mauritania in Africa.

During March 2015–December 2016, we conducted a longitudinal study in the Hospital Center of Atar, situated in the regional capital of Adrar Province, northern Mauritania (Figure 1). Atar is the largest oasis city located in the Sahara, ≈440 km to the northeast of Nouakchott, the national capital. According to the latest census of population and housing, the population of Atar in 2013 was 38,803, primarily Moors (*1*). Rainfall in Atar is scarce (annual mean 50 mm; Office National de la Météorologie, Nouakchott, pers. comm., 2018 Feb 4). During the study period, the total amount of rainfall was 79.2 mm in 2015 and 65 mm in 2016. The average temperature during the same period was 29.5°C (range 23°C–36°C), and the mean relative humidity was 37.1% (range 20%–54%). Date palm culture is the main agricultural activity in Adrar Province; ≈1,200,000 palm trees are distributed across 75 oases (*4*). Livestock largely consists of dromedary camels (*Camelus dromedarius*) and small ruminants (goats and sheep).

### Inclusion Criteria, Sample, and Data Collection

The study included all febrile patients in whom malaria infection was suspected, with either a measured body temperature >37.5°C at the time of consultation or history of fever within the previous 48 hours with no other obvious cause (*5*). After obtaining informed consent, we obtained finger-prick blood samples from patients to prepare thick and thin smears and perform rapid diagnostic testing for malaria. About 100 µL of capillary blood was spotted and dried on Whatman 3MM filter paper (GE Healthcare Europe GmbH, https://www.gehealthcare.com) for PCR diagnosis.

During the consultation, we interviewed patients by using a standard, pretested questionnaire covering sociodemographic data, including detailed recent travel history outside the region and bed net use. We classified frequency of bed net use as always, often, seldom, or never.

### Malaria Detection Methods

#### Rapid Diagnostic Test 

We used Bioline Malaria Antigen Pf/Pan test (Standard Diagnostics/Abbott, https://www.abbott.com) to screen malaria-infected patients. This rapid diagnostic test (RDT) detects *P. falciparum*–specific histidine-rich protein 2, referred to here as the Pf band, and *Plasmodium* genus–specific lactate dehydrogenase, referred to as the Pan band. RDT results were blinded with regard to microscopic examination and PCR results.

#### Microscopic Examination

We dried blood films and stained them with 5% Giemsa solution for 20 min and examined them for the presence of malaria parasites. We counted the number of asexual parasites against 200 leukocytes and expressed parasite density as the number of asexual parasites/µL of blood, assuming a leukocyte count of 8,000/μL of blood (*6*). We recorded the average count of 2 experienced technicians as the final parasite density. We considered a thick blood film as negative if no asexual stage of *Plasmodium* spp. was found after an examination of 100 fields under oil immersion at a magnification of ×1,000. An experienced microscopist performed quality control by reexamining all positive samples and 10% of negative blood smears blindly.

#### PCR

We extracted parasite DNA by using the Chelex method (*7*). We identified *Plasmodium* species by using a species-specific nested PCR targeting mitochondrial cytochrome *b* gene (*8*).

### Statistical Analysis

We initially entered data into an Excel 2003 spreadsheet (Microsoft, http://www.microsoft.com). We evaluated performance (sensitivity, specificity, positive and negative predictive values, and accuracy) of 2 diagnostic tools (microscopic examination and RDT) against PCR as the reference method by using MedCalc statistical calculator software (https://www.medcalc.org/calc/diagnostic_test.php). We used the Cohen κ statistic to estimate the degree of agreement (*9*–*11*), classified as follows: <0, very poor; 0–0.20, poor; 0.21–0.40, fair; 0.41–0.60, moderate; 0.61–0.80, good; and >0.80, very good. We used a χ^2^ test or Fisher exact test to compare proportions or to test association between qualitative variables. We calculated odds ratios and CIs by using the fisher.test function of the R statistical software package (*12*). We measured associations between quantitative variables by using the Kendall rank correlation τ with cor.test function in R. We implemented comparison of true positive and false negative parasitemia diagnosed with RDT by using the Mann-Whitney-Wilcoxon test (wilcox.test function). We determined CIs of binomial proportion by using the binom.test function in R (*12*). For all statistical tests, we considered p<0.05 significant.

### Ethics Considerations

The study protocol was reviewed and approved by the Institutional Ethics Committee of the Université de Nouakchott Al-Aasriya and the Institutional Ethics Committee of the Institut de Recherche pour le Développement, Marseille, France. We obtained written informed consent from adult patients or the parents or legal guardians of children.

## Results

### General Characteristics of the Study Population

We screened a total of 453 febrile patients (235 in 2015 and 218 in 2016; male-to-female sex ratio 1.2) for malaria parasites (Table 1). The mean age of the participants was 29.2 (+18.3 SD) years (range 3 months–80 years; median 27 years). The largest age group was persons >20 years of age (65%). The mean axillary temperature at the time of consultation was 39.2°C (+0.7°C). At the time of consultation, 448 (87%) patients had fever (axillary temperature >37.5°C). Most of the study population were white Moors (77.7%) and black Moors (19.6%). Ten (2.2%) patients were black Africans (i.e., persons of Pular [also known as Peul] and Soninke ethnicity), and 2 (0.4%) patients were foreign expatriates. Almost all patients (441/453 [97.3%]) were residents of Atar and the neighboring oases.

**Table 1:** Demographic characteristics of malaria study population, Atar, northern Mauritania, 2015–2016

Characteristic	No. (%) patients		
	2015	2016	2015–2016
Sex			
F	111 (47.2)	98 (45.0)	209 (46.1)
M	124 (52.8)	120 (55.0)	244 (53.9)
Age group, y			
<5	18 (7.7)	14 (6.4)	32 (7.1)
5–9	14 (6.0)	24 (11.0)	38 (8.4)
10–14	19 (8.1)	19 (8.7)	38 (8.4)
15–19	33 (14.0)	17 (7.8)	50 (11.0)
≥20	151 (64.3)	144 (66.1)	295 (65.1)
Ethnicity			
White Moors	191 (81.3)	161 (73.9)	352 (77.7)
Black Moors*	37 (15.7)	52 (23.9)	89 (19.6)
Black Africans	5 (2.1)	5 (2.3)	10 (2.2)
Foreigners†	2 (0.9)	0	2 (0.4)
Total	235 (100)	218 (100)	453 (100)

*Comparison between 2015 and 2016 showed that only the proportion of Black Moors was statistically significant (p<0.05). The term black Africans refers to ethnic groups (Soninke and Pular [also known as Peul]) of African origin in Mauritania. †Includes 1 expatriate from India and 1 from Mali (Bambara ethnicity).

### Malaria Prevalence and *Plasmodium* Species

Of 453 enrolled patients, 108 (23.8%) were positive for malaria by RDT, 154 (34.0%) were positive by microscopic examination, and 162 (35.8%) were positive by PCR (Table 2). *P. vivax* was by far the dominant species. Among 108 RDT-positive patients, 92 (85.2%) were non–*P. falciparum* and 16 (14.8%) were “*P. falciparum* present” (i.e., infected with *P. falciparum*, with or without non–*P. falciparum*). PCR confirmed that all non–*P. falciparum* cases were attributable to pure *P. vivax*, except in 3 patients (1 with pure *P. falciparum* infection, 1 with *P. falciparum*–*P. vivax* mixed infection, and 1 who was negative for *Plasmodium* spp). *P. ovale* was detected in 10 (6.5%) of 154 patients found to be positive by microscopic examination, but PCR results showed that these parasites were all pure *P. vivax* infections. Among 162 PCR-positive cases, 120 (74.1%) were attributable to pure *P. vivax*, 4 (2.5%) to pure *P. falciparum*, and 38 (23.4%) to *P. falciparum*–*P. vivax* mixed infections.

**Table 2:** Proportions of malaria-positive results and *Plasmodium* species identified among 453 febrile patients, by diagnostic method, Atar, northern Mauritania, 2015–2016*

*Plasmodium* spp.	Microscopic examination	PCR
*P. vivax*	140 (90.9)	120 (74.1)
*P. falciparum*	4 (2.6)	4 (2.5)
*P. falciparum*–*P. vivax*	0	38 (23.4)
*P. ovale*	10 (6.5)	0
Proportions of positive results	154/453 (34.0)	162/453 (35.8)

*Results are expressed as the number of malaria-positive samples and the proportions of malaria species among 154 samples positive by microscopic examination and 162 samples positive by PCR. Proportions of positive results denote the number of malaria-positive samples among all tested samples (n = 453). Rapid diagnostic tests were positive in a total of 108/453 (23.8%) patients and detected 92/108 (85.2%) non–*P. falciparum* only (i.e., *P. vivax*, *P. ovale*, and/or *P. malariae*) and 16/108 (14.8%) *P. falciparum* present (*P. falciparum* with or without *P. vivax*). PCR did not detect any *P. ovale* or *P. malariae*.

### Performance of RDT and Microscopic Examination Compared with PCR

We compared the performance of RDT and microscopic examination for the diagnosis of malaria using PCR as the reference method. Of 162 cases positive by PCR, 55 (33.9%) were negative by RDT, and of 291 negative by PCR, 1 (0.3%) was positive by RDT. In terms of RDT detecting “non–*P. falciparum* only” infections, sensitivity was 63.3%, specificity was 99.3%, positive predictive value was 98.0%, and negative predictive value was 83.5% (Table 3). In comparison, microscopic examination had 82.9% sensitivity, 96.9% specificity, 93.6% positive predictive value, and 91.4% negative predictive value for detecting *P. vivax*. In terms of RDT detecting any *Plasmodium* species, sensitivity was 66.0% and specificity was 99.7%. Of 162 PCR-positive cases, 14 (8.6%) were negative by microscopic examination, and of 291 PCR-negative cases, 6 (2.1%) were positive by microscopic examination. In comparison, microscopic examination had 91.4% sensitivity and 97.9% specificity in detecting any *Plasmodium* species. 

**Table 3:** Performance of rapid diagnostic testing and microscopic examination in establishing malaria diagnosis in 453 febrile patients, using PCR as reference standard, Atar, northern Mauritania, 2015–2016*

Performance	% (95% CI)†						
	Rapid diagnostic test				Microscopic examination		
	Pv	Pf	Pv–Pf		Pv	Pf	Pv–Pf
Sensitivity	63.3 (55.3–70.8)	28.6 (15.7–44.6)	66.0 (58.2–73.3)		82.9 (76.1–88.4)	4.8 (0.6–16.2)	91.4 (85.9–95.2)
Specificity	99.3 (97.6–99.9)	99.0 (97.5–99.7)	99.7 (98.1–99.9)		96.9 (94.3–98.6)	99.5 (98.3–99.9)	97.9 (95.6–99.2)
PPV	98.0 (92.6–99.5)	75.0 (50.3–89.9)	99.1 (93.8–99.9)		93.6 (88.4–96.5)	50.0 (12.6–87.4)	96.1 (91.8–98.2)
NPV	83.5 (80.4–86.1)	93.1 (91.8–94.3)	84.1 (81.0–86.7)		91.4 (88.2–93.7)	91.1 (90.5–91.6)	95.3 (92.5–97.1)
Accuracy	86.8 (83.3–89.7)	92.5 (89.7–94.7)	87.6 (84.2–90.5)		92.1 (89.2–94.4)	90.7 (87.7–93.2)	95.6 (93.3–97.3)

*NPV, negative predictive value; Pf, pure *P. falciparum* plus mixed *P. falciparum–P. vivax*; PPV, positive predictive value; Pv, pure *P. vivax* plus mixed *P. falciparum–P. vivax*; Pv–Pf, pure *P. vivax* plus pure *P. falciparum* plus mixed *P. falciparum–P. vivax*. †Percentages of PCR-positive patients (i.e., the percentage of positives for different *Plasmodium* spp. among included patients [n = 435]), were as follows: 34.9% (95% CI 30.5%–39.5%) for pure *P. vivax*; 9.3% (95% CI 6.8%–12.3%) for pure *P. falciparum*; and 35.8% (95% CI 31.3%–40.4%) for *P.* vivax–*P. falciparum* mixed infections. PCR showed that none of the samples had *P. ovale* or *P. malariae*.

The sensitivity of RDT and microscopic examination was low in our study, mainly because of low parasitemia levels. Overall, regardless of the different plasmodial species, parasitemia was significantly higher in true positives than in false negatives diagnosed by RDT (p = 2.6 × 10^–8^). Parasite density among *P. vivax*–infected patients ranged from 12 to 84,800 asexual parasites/µL of blood with a geometric mean of 1,450 parasites/µL of blood. Among the 100 true positives detected by RDT, the geometric mean parasitemia of *P. vivax* with or without *P. falciparum* was 2,410 asexual parasites/µL (range 12–84,800 asexual parasites/µL), and 11 were negative by microscopic examination. By contrast, the geometric mean parasitemia among the 58 false negatives was 251 asexual parasites/µL (range 40–2,840 asexual parasites/µL), and 5 were negative by microscopic examination. As for 6 cases of *P. falciparum* only infection according to RDT (i.e., having a positive Pf band and negative Pan band), parasitemia ranged from 40 to 440 asexual parasites/µL (n = 3) or was negative (n = 3) by microscopic examination. Among RDT-positive cases with a positive Pf band and positive Pan band (*P. falciparum* with or without non–*P. falciparum*), 6 were negative by microscopic examination and 3 had relatively low parasitemias (<2,000 asexual parasites/µL). Furthermore, parasite density was not significantly correlated with the age of patients (r = 0.03; p = 0.28). We observed very good agreement between microscopic examination and PCR (κ coefficient 0.95 [95 CI% 0.92–0.98]), good agreement between RDT and PCR (κ coefficient 0.72 [95 CI% 0.65–0.78]), and moderate agreement between microscopic examination and RDT (κ coefficient 0.61 [95% CI 0.54–0.69]).

### Seasonality of Malaria

**Figure 2:**
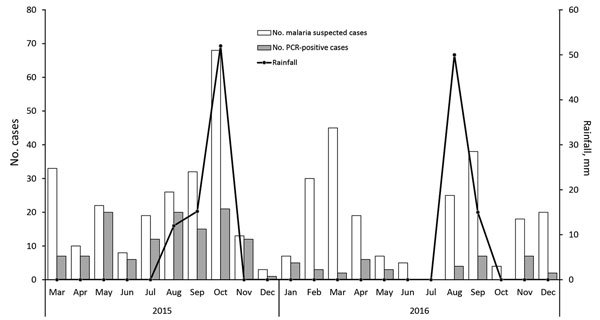
Frequency and monthly distribution of malaria cases diagnosed by using PCR and rainfall amounts in Atar, northern Mauritania, 2015–2016.

PCR-positive malaria cases were detected throughout the study period, except in June, July, and October 2016, during which no malaria cases were detected (Figure 2). In 2015, malaria cases were detected more frequently than in 2016 (121 cases in 2015 vs. 42 in 2016). Although rainfall occurred for 3 months (August–October) in 2015 and 2 months (August–September) in 2016, we observed no correlation between amount of rainfall and monthly cases of malaria (p>0.05).

### Ethnicity, Travel History, and Bed Net Use

Malaria cases were observed in all ethnic groups, including *P. vivax* in 6 black Africans (Table 4). Travel history was reported by 202/435 (46.4%) febrile patients (233 patients had no history of travel and data were missing for 18 patients). Of 162 PCR-positive malaria patients, travel history was available for 151, of whom 48 (31.8%) reported they had never traveled outside Atar and the neighboring oases, 10 (6.6%) had traveled to non–malaria endemic northern Saharan regions of the country, and 93 (61.6%) had a travel history in malaria-endemic regions within the previous 6 months (Table 5). Of the 93 patients with a travel history to malaria-endemic regions, 55 (59.1%) patients (48 with *P. vivax* infection and 7 with *P. falciparum–P. vivax* mixed infections) stayed recently in Nouakchott. Twenty of 42 (47.6%) patients infected with *P. falciparum* with or without *P. vivax* had a travel history to malaria-endemic areas (including 2 patients who traveled to Côte d’Ivoire) within 6 months before consultation, whereas 6/42 patients had missing data on travel history. Sixteen PCR-positive *P. falciparum*–infected patients (either pure [n = 3] or *P. falciparum–P. vivax* mixed [n = 13] infections) occurred in patients who had never traveled outside Atar and the neighboring oases; 2 patients were 5–9 years of age, 2 were 10–15 years of age, and 12 were >15 years of age. 

**Table 4:** Malaria prevalence in 453 febrile patients, by ethnicity, Atar, northern Mauritania, 2015–2016

Ethnicity	No. patients	PCR-positive, no. (%)	*Plasmodium* spp., no. (%)		
			*P. vivax*	*P. falciparum*	Mixed infection*
White Moors	352	129 (36.6)	96 (74.4)	4 (3.1)	29 (22.5)
Black Moors	89	26 (29.2)	19 (73.1)	0	7 (26.9)
Black Africans	10	6† (60.0)	5 (83.3)	0	1 (16.7)
Foreigners	2‡	1 (50.0)	0	0	1 (100)
Total	453	162 (35.8)	120 (74.1)	4 (2.5)	38 (23.5)

*P. falciparum–*P. vivax* infection. †The term “black Africans” refers to ethnic groups (Soninke and Pular [also known as Peul]) of African origin in Mauritania. These 6 patients include 4 persons of Pular ethnicity and 2 persons of Soninke ethnicity. ‡Includes 1 expatriate from India and 1 from Mali (Bambara ethnicity).

**Table 5:** Travel history of malaria-infected patients, by age group, Atar, northern Mauritania, 2015–2016*

Age group, y	No. PCR-positive patients			Total no. (%)
	No travel history	Travel to nonendemic regions	Travel to endemic regions†	
<5	2	0	5	7 (4.6)
5–9	3	0	0	3 (2.0)
10–15	8	0	12	20 (13.2)
>15	35	10	76	121 (80.1)
Total, no. (%)	48 (31.8)	10 (6.6)	93 (61.6)	151 (100)

*Travel history within 6 months before consultation was obtained for 435 febrile patients. Of 162 PCR-positive malaria patients, travel history was available for 151 patients. †Endemic regions include Nouakchott, the entire southern Sahelian zone of Mauritania, and sub-Saharan Africa countries.

Information on bed net use was obtained from 305 patients, 104 (34.1%) of whom had PCR-confirmed malaria. None of the febrile patients in the always group had malaria. The frequent use of bed nets (observed in the always and often groups in contrast with the seldom and never groups) protected against malaria infection (odds ratio 0.31 [95% CI 0.17 – 0.54]; p = 0.00001).

## Discussion

Northern Mauritania is part of the Sahara, the great desert that lies between sub-Saharan Africa, where *P. falciparum* malaria is highly endemic, and the northernmost zone along the Mediterranean Sea, where malaria was eliminated decades ago. In the Sahara, oases are the main agro-ecologic environment suitable for malaria transmission. In Mauritania, the number of oases in the Adrar region increased by 140%, from 31 in 1984 to 75 in 2012, because of the development of hydro-agricultural projects (*4*). These oases are also the site of increasing tourism by thousands of Mauritanians who visit the Mauritanian Adrar every year during June–August for Guetna, the Arabic name for the season when date palm fruit are harvested, and the return of foreign tourists, particularly from Europe, after a 10-year collapse in tourism. Furthermore, oases in the Sahara constitute a transit zone for thousands of migrants from sub-Saharan Africa on their way to Maghreb countries (countries in North Africa bordering the Mediterranean Sea) and Europe.

Despite the potential risk for malaria transmission, limited data are available on oasis malaria in the Sahara (*13*–*17*). In our study, the predominant *Plasmodium* species found in the population was *P. vivax*, which affected persons of the major ethnic groups present in the country. This finding is consistent with the results of earlier studies conducted in Nouakchott, the capital city of Mauritania situated in the Sahara (*18*,*19*). Those studies showed that most *P. vivax* malaria–infected patients were Duffy-positive white Moorish persons. In our study, we also observed *P. vivax* infections among malaria-positive patients of black Moorish and black African ethnicities. Many recent reports, particularly from sub-Saharan Africa countries and Madagascar, also showed that *P. vivax* can infect Duffy-negative black African persons, although at low frequencies (*20*–*22*). The existence of a second distinct *P. vivax* erythrocyte-binding protein, which most likely mediates parasite invasion of the reticulocyte, was recently suggested (*23*).

The origin of *P. vivax* in Mauritania, particularly in the Saharan zone, is not yet clear. Several foci of *P. vivax* were present in parts of Morocco, a country north of Mauritania, but malaria was eliminated from Morocco in 2010 (*24*). Active foci of *P. vivax* malaria transmission still exist in Algeria, albeit at a very low prevalence (*13*), and recent reports suggest that the goal of malaria elimination might soon be achieved in Algeria (*2*). Recently, *P. vivax* infections were reported from Mali and Senegal, 2 neighboring countries to the east and southeast of Mauritania, in symptomatic patients and in asymptomatic children (*22*,*25*,*26*). More surprisingly, recent evidence showed that *P. vivax* is present in some countries of central Africa, such as Cameroon (*21*), and that extensive molecular investigations might show that *P. vivax* prevalence in Africa is much higher than previously assumed (*27*,*28*). Elsewhere in eastern Africa, such as Sudan (*29*), Ethiopia (*30*), and Madagascar (*20*), *P. vivax* malaria has been known to exist.

We report that *P. falciparum* also has been diagnosed among febrile patients permanently residing in Atar, albeit at a relatively low rate, a finding that is consistent with previous studies on the geographic distribution of *Plasmodium* species in Mauritania (*31*–*34*). However, the data in our study suggest that *P. falciparum* might have been introduced to Atar by travelers visiting malaria-endemic regions to the south. As far as *P. falciparum* in Atar is concerned, further studies are required to establish whether local transmission of this *Plasmodium* species occurs. It is worth noting that autochthonous *P. falciparum* malaria was already reported in a village in the Algerian Sahara near the Algeria–Mali border (*35*).

The sensitivity of RDT and microscopic examination was low in our study. For *P. vivax*, *Plasmodium* genus–specific lactate dehydrogenase RDT has low sensitivity to detect this parasite species at low parasitemia levels (*36*). Relatively few patients were infected with *P. falciparum* with or without *P. vivax*, and these infections were rarely diagnosed correctly by laboratory technicians, which can lead to problems in the management of symptomatic patients, even with the use of RDT in case of low parasitemia levels. Microscopic examination also misdiagnosed 10 samples as *P. ovale*. PCR showed that these cases were actually *P. vivax* or mixed *P. vivax*–*P. falciparum* infections. *P. ovale* and *P. vivax* are notoriously difficult to distinguish morphologically by microscopic examination, and accurate identification requires an experienced microscopist, relevant clinical information, and up-to-date epidemiologic data. PCR findings in our study further confirm the results of earlier studies conducted in Nouakchott and some cities in southern Mauritania, indicating that *P. ovale* has not yet been detected by PCR in Mauritania (*3*,*31*).

In areas of intense *P. falciparum* transmission, parasite density and age are negatively correlated; children <5 years of age are generally infected with higher parasite density than older children and adults (*37*). In our study, no significant correlation was observed between *P. vivax* parasite density and the age of malaria patients. Similar findings were reported from studies conducted in the Malian Sahara (*14*,*16*), suggesting unstable malaria transmission in which adults do not acquire protective immunity because of a lack of continuous exposure to infective bites of the *Anopheles* mosquito vectors. In Atar, malaria cases were reported throughout the year. At least some of these *P. vivax* cases might be attributable to recurrent infections that result from reactivation of hypnozoites in the liver, which are considered important contributing factors to disease in *P. vivax* infections, accounting for as many as 50% of *P. vivax* infections in some reports (*38*,*39*). Alternatively, these cases might be primo-infections and *P. vivax* transmission occurs throughout the year. Autochthonous transmission does occur in Atar, at least during some periods of the year, evidenced by the high number of PCR-confirmed malaria-infected patients who did not report any travel history.

No entomologic evidence exists to indicate that malaria transmission occurs in the oasis setting of Atar. Nevertheless, the protection provided by regular use of bed nets supports the hypothesis that local transmission is occurring. Moreover, the presence of numerous natural water bodies (e.g., rain puddles, small and large ponds locally known as Guelta, and small brooks) and artificial water bodies (e.g., shallow wells, open-pit cement containers, and palm irrigation channels) might serve as breeding habitats for *Anopheles* mosquitoes. However, water in these potential habitats is generally brackish and often covered with filamentous algae (*40*). During the study period, the *An. rhodesiensis* mosquito was the only *Anopheles* mosquito species collected during both larval and adult surveys (K. Mint Lekweiry, unpub. data). The vector capacity of *An. rhodesiensis* mosquitoes in Atar is not yet established. The *An. rhodesiensis* mosquito is considered a secondary malaria vector despite sporadic reports of sporozoite positivity in several geographic areas before 1950 (*41*,*42*). However, its capacity to sustain the development and propagation of *Plasmodium* spp. cannot be ruled out. Currently available data suggest that the northernmost range limits of the major continental Africa vector, the *An. arabiensis* mosquito*,* in Mauritania is Rachid oasis in the province of Tagant, located ≈250 km south of Atar (*43*). Thus, concern that the recently constructed national road that connects Rachid oasis to Atar might increase the risk for introducing *An. arabiensis* mosquitoes or other malaria vectors in Atar is warranted.

Although characterized by extreme aridity and low rainfall, *P. vivax* and, to a much lesser extent, *P. falciparum* malaria are present in Atar. Because our data were obtained from a hospital-based study and the numbers of asymptomatic carriers and symptomatic malaria-infected patients who self-medicate with antimalarial drugs or consult the private sector are unknown, the actual malaria burden is probably underestimated. Further entomologic and parasitologic studies are needed to assess the vector capacity of *An. rhodesiensis* mosquitoes and identify the main malaria vector (or vectors) in Atar. Moreover, regular monitoring of malaria in the Saharan zone, including in other oases, should be implemented.
